# Review of Editorial Remarks upon “Sponge Gold” by Dr. J. D. White

**Published:** 1855-01

**Authors:** 


### REVIEW OF EDITORIAL REMARKS UPON "SPONGE GOLD," BY DR. J. D. WHITE.

[See "Dental News Letter," Vol. 7, Page 238 & 9.]

BY J. T.

Somebody has taken the liberty to interrogate Dr. J. D. White, in regard to "Sponge Gold." The Doctor refers to the opinions of others upon the subject; and then proceeds to give his own experience, and theories, in regard to the material in question; and also some peculiar opinions of his upon the subject of filling.

Ever since we first saw this editorial of Dr. W's., we have experienced a kind of disposition to —— oh no, not to give our opinion, but to ask some questions in regard to those theories.

The Dr. *may* be able to establish them, but he has not done it; and we are somewhat in the *fix* of the individual, who concluded he "must have heard a very *wise* sermon, for he did not understand a word of it."

The Dr. says, "That Sponge Gold will weld, and roll into plate, does not make it *necessarily* better than foil, for the "reason that packing it into a cavity and rolling it out into plate are two things." Now, if it *will* weld into a solid mass, is it not better than foil, that will not do so? If that is true, is it not *necessarily* better than a material that will not weld? What are we to understand by the language, "for the reason that packing it into a cavity, and rolling it into plate are two things?" Well, what of it, is that any evidence that it cannot be welded into a solid mass in a cavity?"

Now, what is the principle upon which Sponge or Crystal Gold is united into a solid mass; certainly by *pressure*, both in the mill and in the tooth; and it can be about as perfectly accomplished in the latter as the former. But to accomplish this, the operator must understand the proper method of doing it.

It *is* true, that if a "cavity is filled with a mass" or one piece, and consolidated from the surface, it would be a failure.

Who would attempt to put in a filling in that manner? He condemns any other method of introducing it because "if used in small particles it requires too long a time to fill a cavity. Now the Dr. takes two extremes, either of which if he follows, he cannot make a good filling, with any material or preparation. Does he introduce his foil "into the cavity in a mass" or "in small particles?" Most certainly a sponge, or crystal gold plug, when consolidated, *is* "much more solid than a foil plug" can be made.

Well, really we should be pleased to see the rationale of "the dampness of the mouth or the tooth breaking up the texture of the plug," and causing it to crumble out." What is the theory upon which "dampness" causes gold that is welded into a solid mass to crumble to pieces, to become disintegrated? Is "dampness" a solvent for gold? *Magnificent!* But it must be so, the Doctor says "We have had this to occur with us in many instances, and it has also occurred to others."—Astonishing. Who has yet found a material for filling teeth that failures did not "occur," and to those who were much in favor of its use too?

In all those cases of breaking up of the texture of the plug, there *was a failure* to consolidate properly, or the sponge or crystal was not good of its kind. "We have had on several occasions to remove plugs of this preparation, on account of the teeth giving trouble after they were plugged."

Oh yes, such fillings always do give trouble, "*double trouble*," if the operator has any conscience, they first trouble the operator, and afterwards the patient. The Doctor does not tell us which in these cases.

But the fact is, these fillings were never made solid. We are told "it was the internal portion of the plug that had become softened." The surface retaining the solid finish that was given to it. This, tho' not expressed, is the legitimate deduction from the language; this dense surface had maintained its position, otherwise it would have fallen out. Now is it in accordance with science or common sense, either to assert that a mass, that was made solid, "had become soft," granulated or disintegrated, and yet not occupy more space than when dense.

It was not made solid at first. If it was, and the dampness or moisture softened it, why did not the surface of the filling become soft first. The fact that the surface did retain its density, even when continually subject to the influence of moisture, is conclusive evidence that the internal part had not been consolidated; and moreover, that it was susceptible of being united into a solid mass, that would not be affected by moisture, even when it could come in contact with it, and much less when it could not.

"We have plugged some large cavities on the buccal surfaces of the molar teeth, and polished them well, and made them hard as we knew how, and we can apply as much force as almost any other dentist, having a goodly share of muscular strength."

Now Doctor, we did suppose that you were aware, long before this, that perfect execution in the operation of filling teeth does not depend upon the great amount of "muscular strength" possessed by the operator; does it not depend more upon the skill of the operator, and the perfect form of his instruments, than upon muscular strength? Just imagine a strong robust man filling a delicate lady's tooth with a force of from one to two hundred pounds; you could apply more force than that could you not?

"And applied the plugging forceps, and cut them to pieces." Most certainly you could if put in, in the manner you have described.

"It is believed by some, that a filling must be *impervious* to *dampness*." Well, yes, everybody that we ever heard express an opinion, and all writers except yourself, entertain the same belief.—"This it cannot be, if it were, it would not be necessarily a perfect plug." Now Doctor do you intend to say, that a plug that will perfectly exclude the dampness is an imperfect plug *because* it excludes it; I hope you have some *way* of explaining this that is not in the language.

"Dampness must penetrate a plug to some extent, or the dampness will force around the plug, and displace it, sooner or later."

Here is that rascally old chap *dampness*, again; a while ago, he played the mischief by getting into the plug, and now he is going to kick up the *dust* if he don't get in. What? why, he will *pull* or *push* our plugs out "*sooner* or *later*." Persevering old chap that.

Are we to understand that moisture must permeate a plug to *some* extent? Well, to what extent? If it permeates to *some* extent, it *will* permeate through the entire mass of the plug, and if so, it must come in contact with the walls of the cavity, and caries would follow, and away would go your soft plug. But upon what principle is it that the moisture will be forced around the plug if it can not penetrate it? Where is the forcing power? is it an inherent property of the moisture? and why will it not force around a plug that it can permeate, and displace it, just as readily as that it can not permeate? Doctor, we are in the dark on some of these points. Please enlighten.

"We know well, that a distinguished operator in our city loses more hard plugs than soft ones, on that account."

On *what* account? For what is he distinguished? For losing more hard plugs than soft ones—*on that account?* Oh, yes, we have some operators here who are *distinguished* for *that* too. They lose *all* their *hard* plugs, and what is worse, all their hard plugs are soft—freaks of dampness.

His plugs are *therefore*"—[what a place for a therefore]—"better than the teeth he puts them in. Why? Oh, because they come out; Of course, the sooner they come out the better plugs they are—*therefore.* Taking this for a criterion we *wot* of some who make most excellent plugs.

The Doctor is unfortunate in his logic. It is customary to have some connection between the premises and the conclusion, but in this case they are so entirely independent of, and separate from one another, that if the one had the small-pox, the other would be perfectly free from danger, without vaccination.

"We do not wish to be understood as not advocating hard plugs." Well, Doctor, if you are advocating hard plugs, you do it in a very singular manner. You say that a plug, so dense as to *exclude dampness*, is not necessarily a perfect plug; and yet *you* advocate hard plugs. "A distinguished operator loses more hard plugs than soft ones." And yet *you* advocate hard plugs. "We believe the best plug is one that is of about equal porosity to the dentine." How are we to make a filling in a tooth "of equal porosity to the dentine, with a material so unlike dentine as gold. Dentine is porous, gold is not. A gold plug, even when soft, is not porous in the same manner as dentine. How are we to make two things of general porosity, so unlike in their nature as dentine and gold? Did you intend density, then you are equally unfortunate, for it is impossible to make a plug of gold in a tooth, of similar density to the dentine, because of the different nature of the material. Well, did you intend, of equal hardness to the dentine? This you could not mean, for you *know* you could not make a gold plug as hard as dentine, and most certainly you do not make your plugs as hard as you could.

"No reasonably good operator loses a plug by softening, but by the margins of his cavity giving away? not because the plug was too hard, but because it was not consolidated perfectly against the walls of the cavity; and permitted the ingress of the secretions of the mouth and the subsequent decay of the walls of the cavity.

"The constant expansion and contraction of the plug and the tooth will cause any plug to give way sooner or later."

Is the contraction and expansion of the tooth and plug exhibited by opposite principles; or will they not both expand under the influence of increased temperature, and both contract under diminished temperature?

Doctor, will you be so kind as to give us the amount of expansion of a tooth and also of a gold plug, of any given size, under the influence of the variations of temperature to which they are exposed in the mouth, and what will be the relative difference of expansion?

And if this difference really does exist, would not the texture of a soft plug be broken up much more readily thereby than a hard one? It would be rather a comical operation to attempt to disintegrate or break up the texture of a piece of gold by a uniform pressure upon all its sides, or all but one; we would expect it would require some time to do it, even with the aid of "*dampness.*"

